# Theranostic Nanomedicine for Synergistic Chemodynamic Therapy and Chemotherapy of Orthotopic Glioma

**DOI:** 10.1002/advs.202003036

**Published:** 2020-11-13

**Authors:** Junyi Tan, Xiaohui Duan, Fang Zhang, Xiaohua Ban, Jiaji Mao, Minghui Cao, Shisong Han, Xintao Shuai, Jun Shen

**Affiliations:** ^1^ Department of Radiology Sun Yat‐sen Memorial Hospital Sun Yat‐sen University Guangzhou 510120 China; ^2^ PCFM Lab of Ministry of Education School of Materials Science and Engineering Sun Yat‐sen University Guangzhou 510275 China; ^3^ Guangdong Provincial Key Laboratory of Malignant Tumour Epigenetics and Gene Regulation Sun Yat‐sen Memorial Hospital Sun Yat‐sen University Guangzhou 510120 China; ^4^ Department of Radiology Sun Yat‐sen University Cancer Centre Sun Yat‐sen University Guangzhou 510060 China

**Keywords:** chemodynamic therapy, chemotherapy, glioma, magnetic resonance imaging, tumor microenvironment

## Abstract

Glioma is a common primary brain malignancy with a poor prognosis. Chemotherapy is the first‐line treatment for brain tumors but low efficiency of drugs in crossing the blood–brain barrier (BBB) and drug resistance related to tumor hypoxia thwart its efficacy. Herein, a theranostic nanodrug (iRPPA@TMZ/MnO) is developed by incorporating oleic acid‐modified manganese oxide (MnO) and temozolomide (TMZ) into a polyethylene glycol‐poly(2‐(diisopropylamino)ethyl methacrylate‐based polymeric micelle containing internalizing arginine‐glycine‐aspartic acid (iRGD). The presence of iRGD provides the nanodrug with a high capacity of crossing the BBB and penetrating the tumor tissue. After accumulation in glioma, the nanodrug responds to the tumor microenvironment to simultaneously release TMZ, Mn^2+^, and O_2_. The released TMZ induces tumor cell apoptosis and the released Mn^2+^ causes intracellular oxidative stress that kill tumor cells via a Fenton‐like reaction. The O_2_ produced in situ alleviates tumor hypoxia and enhances the chemotherapy/chemodynamic therapeutic effects against glioma. The Mn^2+^ can also serve as a magnetic resonance imaging (MRI) contrast agent for tumor imaging during therapy. The study demonstrates the great potential of this multifunctional nanodrug for MRI‐visible therapy of brain glioma.

## Graphical abstract

A theranostic nanodrug (iRPPA@TMZ/MnO) exhibiting a high capability of crossing the blood–brain barrier and accumulating in glioma is developed. This nanodrug can respond to the tumor microenvironment to alleviate tumor hypoxia, exert synergistic chemotherapy/chemodynamic therapy, and enable site‐specific magnetic resonance imaging for effective treatment of orthotopic brain glioma.

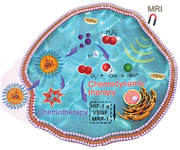

## 1 Introduction

Glioma is the most common malignancy of the central nervous system (CNS), with an overall annual incidence of 5–6 per 100 000 individuals in the United States. The average 5 year survival of patients with glioma is less than 35%.^[^ ^1^ ^]^ At present, maximal surgical resection of tumors combined with radiotherapy and/or systemic chemotherapy is the first choice for treating this fatal disease.^[^ ^2^ ^]^ The local surgical resection cannot entirely eliminate the tumor tissue due to the invasive nature of glioma and the undefined tumor margin. Thus, postsurgical treatment is usually necessary to prevent the recurrence of glioma.^[^ ^2b^ ^]^ Oral alkylating agent temozolomide (TMZ) is recommended as the first‐line drug for systemic chemotherapy against glioma and can be used after surgery or for recurrent tumors.^[^ ^3^ ^]^ Unfortunately, the effectiveness of TMZ for glioma treatment is often transient and limited due to drug resistance.^[^ ^3^ ^]^ There is a clear need to develop more effective therapeutic strategies for glioma treatment.

Hypoxia is a hallmark of the solid tumor microenvironment (TME) that can not only promote cancer cell proliferation and invasion into normal brain tissues but also induce resistance to multiple therapies.^[^ ^4^ ^]^ Recent evidence has indicated that the hypoxia of glioma tissue can result in tumor cell resistance to TMZ through the hypoxia‐inducible factor‐1*α* (HIF‐1*α*) pathway.^[^ ^5^ ^]^ Various methods have been proposed to overcome hypoxic TME including perfluorocarbon‐based oxygen supply,^[^ ^6^ ^]^ hyperbaric oxygen therapy,^[^ ^7^ ^]^ and activation of prodrugs via the hypoxia pathway.^[^ ^8^ ^]^ However, these strategies have shown relatively low efficiency thus far.

Manganese dioxide (MnO_2_) /Manganese oxide (MnO) nanoparticles have recently been used to modulate tumor hypoxia.^[^ ^9^ ^]^ Owing to their high response to pH and high reactivity with hydrogen peroxide (H_2_O_2_) and glutathione (GSH), MnO_2_/MnO delivered to tumor tissue can rapidly react to release Mn^2+^ and O_2_.^[^ ^9^, ^10^ ^]^ The O_2_ released in situ can modulate tumor hypoxia by downregulating the expression of HIF‐1*α* and vascular endothelial growth factor (VEGF), thereby enhancing tumor sensitivity to all therapies including chemotherapy, radiotherapy, reactive oxygen species (ROS)‐related therapy, and immunotherapy.^[^ ^9a,d^ ^]^ The released Mn^2+^ can also serve as a potent T1‐weighted magnetic resonance imaging (MRI) contrast agent to monitor the drug‐delivery process.^[^ ^9d^ ^]^ Compared with the clinically available Gd‐based contrast agents, MnO_2_/MnO‐based nanoparticles only function as MRI contrast agents in TME conditions (low pH, high H_2_O_2_, and high GSH); this can increase the target‐to‐background signal ratio and improve the MRI detection sensitivity and specificity to solid tumors.^[^ ^9^, ^11^ ^]^ In addition to the modulation of tumor hypoxia and TME‐activatable MRI via a Fenton‐like reaction to release O_2_ and Mn^2+^, a recent study has indicated that the Fenton‐like reaction of MnO_2_/MnO nanoparticles in TME can also deplete GSH, disrupt the cellular antioxidant defense system, and generate oxidative hydroxyl (·OH) radicals inside tumor cells to exert an effective antitumor effect.^[^ ^12^ ^]^ These multiple functions make MnO_2_/MnO‐based nanoparticles a promising nanotheranostic platform for clinical applications. To date, most of the MnO_2_/MnO nanoparticles have been synthesized in a water‐soluble form through a reduction reaction. However, such water‐soluble MnO_2_/MnO nanoparticles have drawbacks including toxicity caused by manganese ions leakage and accumulation in normal tissues, ineffective modification due to the naked surface of the oxide, and a broad particle‐size distribution resulting from the hydrothermal reaction that makes the nanoparticles difficult to integrate with other common cargo delivery strategies. To the best of our knowledge, MnO_2_/MnO‐based nanoparticles have been used mainly in the treatment of other solid tumors rather than glioma. In particular, less data have been reported on alleviation of hypoxia and provision of effective chemotherapy against glioma due to the presence and function of the blood–brain barrier (BBB) in the CNS. The BBB is a natural obstacle that prevents almost all nanoscale objects from entering the brain.^[^ ^13^ ^]^ Hence, the development of MnO_2_/MnO‐based nanoparticles capable of crossing the BBB after systemic administration is vital for utilizing the above‐mentioned multiple functions.

**Scheme 1:**
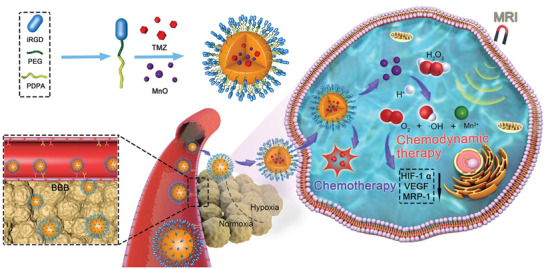
Schematic illustrations of iRPPA@TMZ/MnO for MRI‐guided synergistic chemotherapy/CDT against brain glioma.

In the present study, we synthesized monodispersed nanoparticles of oleic manganese monoxide using a modified solvothermal method, encapsulated the nanoparticles in polymeric micelles (polyethylene glycol‐poly(2‐(diisopropylamino)ethyl methacrylate, PEG‐PDPA) with TMZ, and modified the micelles by internalizing arginine‐glycine‐aspartic acid (iRGD) peptide (iRPPA@TMZ/MnO) (**Scheme** **1**). The iRGD peptide‐containing nanoparticles may penetrate tumor blood vessels and tissue through interactions with *α*_v_ *β*_3_ integrin and neuropilin‐1 (NRP‐1) and cross the BBB to reach the glioma cells.^[^ ^14^ ^]^ These multifunctional iRPPA@TMZ/MnO nanoparticles have been designed to be triggered by the TME of glioma tosimultaneously produce TMZ, Mn^2+^, and O_2_. The released TMZ induces apoptosis of tumor cells while the Mn^2+^ induces intracellular oxidative stress to cause tumor cell death via Fenton‐like activity. In parallel, the O_2_ released in situ alleviates tumor hypoxia and enhances the chemotherapy/chemodynamic therapeutic effect against glioma. Additionally, Mn^2+^ has been shown to act as an MRI contrast agent for monitoring of tumors during the treatment process.

## 2 Results and Discussion

### 2.1 Preparation and Characterization of Nanodrugs

**Figure 1:**
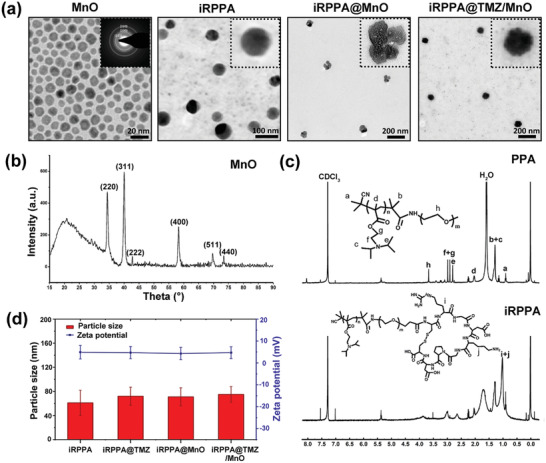
Characterizations of polymer and theranostic nanodrugs. a) TEM images of MnO, iRGD‐PEG‐PDPA (iRPPA), iRPPA@MnO, and iRPPA@TMZ/MnO. b) XRD pattern of MnO. c)^1^H NMR spectra of PEG‐PDPA (PPA) and iRPPA. d) Hydrodynamic diameters and zeta potentials of iRPPA, iRPPA@TMZ, iRPPA@MnO, and iRPPA@TMZ/MnO characterized by DLS.

X‐ray diffraction (XRD) and selected area electron diffraction (SAED) tests were performed to identify the crystalline phase and morphology of the prepared ultrasmall MnO nanoparticles. The well‐defined diffraction peaks were assigned to the (111), (200), (220), (311), and (222) lattice planes indexed to cubic manganosite (JCPDS no.75‐0257). The multicrystalline MnO structure with the corresponding lattice features was further confirmed by electron diffraction of the selected area. Uniform cube‐ or dodecahedron‐shaped high‐contrast crystals, measuring on average ≈15 nm, were observed in the high‐resolution transmission electron microscopy (TEM) image (**Figure** **1**a) that agreed with the result calculated from XRD relative peak intensities using the Scherrer equation (Figure 1b). PEG‐PDPA block copolymer was synthesized by reversible addition–fragmentation chain transfer (RAFT) polymerization. The composition of PEG‐PDPA was determined by ^1^H nuclear magnetic resonance (NMR) analysis. The coupling of the iRGD peptide with the block polymer was confirmed by ^1^H NMR and gel permeation chromatography (GPC) measurements (Figure 1c). The chemical shift around 1.1–1.5 ppm in the ^1^H NMR spectrum indicated the formation of an amide bond between iRGD and the amino group‐terminated PEG block. PEG‐PDPA and iRGD‐PEG‐PDPA (17:3 molar ratio)^[^ ^15^ ^]^ were assembled into micellar nanodrugs with or without loading MnO nanocrystals and/or TMZ into the core via sonification. TEM analysis showed that the blank micelle (iRPPA) had a uniform and relatively small size of 50–60 nm (Figure 1a). From the hysteresis curves, the MnO nanocrystals and MnO‐loaded iRPPA (iRPPA@MnO) nanoparticles at 300 K displayed markedly low saturated magnetization of 6.21 and 10.03 emu g^–1^, respectively (Figure S1, Supporting Information), thereby indicating that both the MnO nanocrystals and iRPPA@MnO nanoparticles were diamagnetic in their original states.

The morphology, size distribution, and zeta potentials of iRPPA and MnO/TMZ‐loaded nanodrugs (iRPPA@MnO, iRPPA@TMZ, and iRPPA@TMZ/MnO) were measured by TEM and dynamic light scattering (DLS) (Figure 1a,d). The hydrodynamic diameter and zeta potential of iRPPA were 61 ± 21 nm and 4.9 ± 3.1 mV, respectively. Encapsulation of MnO and TMZ slightly increased the hydrodynamic diameters but had no effect on the zeta potential of the micelles; the hydrodynamic diameter and zeta potential of iRPPA@TMZ/MnO were 71 ± 15 nm and + 4.3 ± 2.8 mV, respectively. We tested the stability of iRPPA@TMZ/MnO in phosphate buffer saline (PBS, pH = 7.4) and 10% fetal bovine serum (FBS)‐containing PBS mimicking the physiochemical environment, respectively. The particle size of iRPPA@TMZ/MnO in both PBS (pH = 7.4) and 10% FBS‐containing PBS did not obviously change in 14 days, indicating good stability of the nanodrug (Figure S2, Supporting Information). The polydispersity indexes (PIs) of iRPPA, iRPPA@MnO, iRPPA@TMZ, PPA@TMZ/MnO, and iRPPA@TMZ/MnO were 0.192, 0.201, 0.194, 0.234, and 0.231, respectively. As shown in the TEM image (Figure 1a), iRPPA@MnO presented an interesting structure with four MnO nanocrystals in each micellar particle. The entrapment efficiency and loading content of TMZ and MnO in different formulations are listed in **Table** **1**. The loading content of TMZ and MnO in iRPPA@TMZ/MnO was estimated by high‐performance liquid chromatography (HPLC) and inductively coupled plasma‐atomic emission spectrometry (ICP‐AES) and reached 5.9% and 10.2%, respectively.

**Table 1:** The entrapment efficiency and loading content of TMZ and MnO in different formulations

		iRPPA@TMZ	PPA@TMZ	iRPPA@MnO	PPA@MnO	iRPPA@TMZ/MnO	PPA@TMZ/MnO
Entrapment efficiency	TMZ	93.2% ± 0.2%	98.6% ± 0.5%	–	–	80.8% ± 0.5%	86.3% ± 2.0%
	MnO	–	–	65.1% ± 1.2%	95.2% ± 1.6%	60.3% ± 4.2%	66.3% ± 1.9%
Loading content	TMZ	6.8% ± 0.1%	7.2% ± 0.6%	–	–	5.9% ± 0.2%	6.3% ± 0.8%
	MnO	–	–	11.0% ± 0.9%	16.1% ± 1.2%	10.2% ± 2.0%	11.2% ± 0.7%

### 2.2 Responsiveness to Stimulations

**Figure 2:**
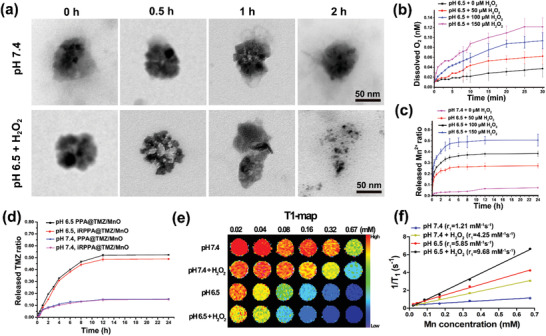
In vitro TME‐responsive properties of nanodrugs. a) Serial TEM images show the decomposition process of iRPPA@TMZ/MnO at pH 7.4 or pH 6.5 + 100 × 10^−6^ mH_2_O_2_. b) Oxygen generation by iRPPA@TMZ/MnO at pH 6.5 + 0 – 150 × 10^−6^ mH_2_O_2_(*n*= 3). c) Mn^2+^produced from iRPPA@TMZ/MnO at pH 7.4 without H_2_O_2_, pH 6.5 + 50 – 150 × 10^−6^ mH_2_O_2_(*n*= 3). d) TME‐responsive drug release profiles of PPA@TMZ/MnO and iRPPA@TMZ/MnO at pH 6.5 and pH 7.4 measured by HPLC (*n*= 3). e) In vitro MRI T1‐map of iRPPA@TMZ/MnO at different stimulations. f) The*r*_1_relaxivities of iRPPA@TMZ/MnO at different stimulations.

The PDPA block of the copolymer has been reported to protonate in a weakly acidic environment;^[^ ^16^ ^]^ this may result in the disassembly of micelles to release the loaded drug in TME. To assess this potential, the TMZ release profile of drug‐loaded micelles in TME‐mimicking conditions was measured by HPLC; 53% of TMZ was released at pH 6.5 in 12 h compared to less than 15% TMZ released at pH 7.4 (**Figure** **2**d). A previous study showed that MnO, that is stable under normal physiological conditions, could be reduced to produce Mn^2+^ and O_2_ in the TME (≈pH 6.5; 100 × 10^−6^ m H_2_O_2_).^[^ ^10^ ^]^ To investigate the decomposition process of iRPPA@MnO and iRPPA@TMZ/MnO, TEM of iRPPA@MnO and iRPPA@TMZ/MnO incubated at pH 7.4 or pH 6.5 with 100 × 10^−6^ m H_2_O_2_ was observed at various time points (Figure 2a; Figure S3, Supporting Information). iRPPA@MnO and iRPPA@TMZ/MnO remained stable at pH 7.4 but they gradually decomposed at pH 6.5 + 100 × 10^−6^ m H_2_O_2_ within 2 h, thereby indicating that iRPPA@MnO and iRPPA@TMZ/MnO might degrade in TME. We further investigated the stimulation‐responsive release of Mn^2+^ and O_2_. As shown in Figure 2b and Figure S3, Supporting Information, iRPPA@TMZ/MnO produced O_2_ quickly, in a concentration‐dependent manner, when incubated at pH 6.5 + 0 – 150 × 10^−6^ m H_2_O_2_. An evident Mn^2+^ release was detected when iRPPA@TMZ/MnO was incubated at pH 6.5 + 50 – 150 × 10^−6^ m H_2_O_2_, whereas negligible Mn^2+^ was produced at neutral pH without H_2_O_2_ (Figure 2c; Figure S3, Supporting Information). These results confirmed that MnO embedded in the nanodrugs could react to produce Mn^2+^ and O_2_ in the TME‐like conditions of low pH and enriched H_2_O_2_.

MnO is known to be a TME‐activatable MRI contrast agent.^[^ ^10^, ^17^ ^]^ The Mn^2+^ produced in TME could exchange with surrounding water molecules much more easily than MnO and could decrease the relaxation times of protons, resulting in an effective MRI enhancement.^[^ ^17^ ^]^ To further evaluate the potential of iRPPA@TMZ/MnO as a TME‐activatable T1‐weighted imaging (T1WI) MRI contrast agent, the signal intensity, longitudinal relaxivity (*r*_1_), and transverse relaxivity (*r*_2_) of this theranostic nanodrug under different stimulations were measured on a 3.0 T MRI scanner (Figure 2e,f; Figures S4 and S5, Supporting Information). The signal intensity gradually decreased on MRI T1‐map and T2‐map along with the increase in Mn concentration. The *r*_1_ and *r*_2_ values of iRPPA@TMZ/MnO were 1.21 × 10^−3^ and 2.40 × 10^−3^ m s^−1^ at pH 7.4 without H_2_O_2_ and increased significantly to 4.25 × 10^−3^, 5.85 × 10^−3^, 9.68 × 10^−3^ m s^−1^, and 5.86 × 10^−3^, 22.93 × 10^−3^, 42.21 × 10^−3^ m s^−1^ at pH 7.4 + 100 × 10^−6^ m H_2_O_2_, pH 6.5 without H_2_O_2_, and pH 6.5 + 100 × 10^−6^ m H_2_O_2_, respectively. The nanodrugs PPA@TMZ/MnO and iRPPA@TMZ/MnO showed similar *r*_1_ and *r*_2_ values under different conditions (Figures S4 and S5, Supporting Information). These results indicated that TME could induce an eightfold increase in *r*_1_ relaxivity when the MnO‐containing nanodrugs were used as a TME‐triggerable contrast agent, thus generating an even better contrast enhancement than the clinically widely used gadolinium‐diethylenetriamine pentaacetic acid (Gd‐DTPA) MRI T1 contrast agent (*r*_1_ = 3.5 × 10^−3^–5.5 × 10^−3^ m s^−1^).^[^ ^18^ ^]^ The abovementioned TME‐triggerable feature may enable iRPPA@TMZ/MnO to act as an MRI contrast agent specific for tumor detection.

### 2.3 In Vitro Cell Studies

**Figure 3:**
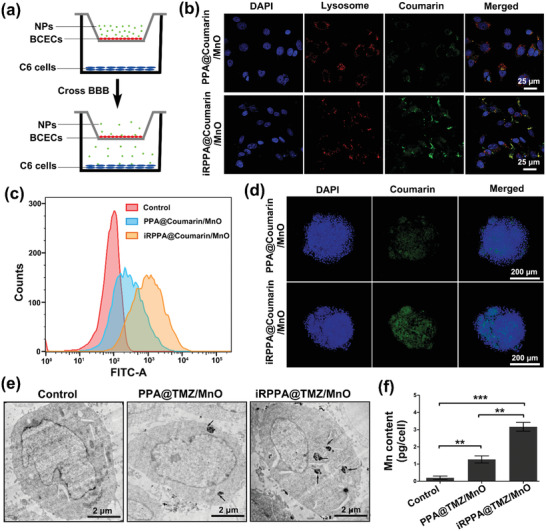
In vitro BBB‐penetrating ability and cellular uptake of nanodrugs. a) An illustration of the in vitro two‐compartment BBB model. b) CLSM and c) quantitative flow cytometric analysis showing the uptake of nanodrugs by C6 glioma cells in the bottom well after incubation with PPA@coumarin/MnO and iRPPA@coumarin/MnO. d) CLSM showing the in vitro penetrating effect of PPA@coumarin/MnO and iRPPA@coumarin/MnO on C6 glioma cell spheroids. e) Representative cellular TEM images of C6 glioma cells treated with PBS, PPA@TMZ/MnO, and iRPPA@TMZ/MnO. Black arrows indicate the uptake of nanoparticle clusters. f) The graph shows the intracellular manganese contents of C6 glioma cells after incubation with PBS, PPA@TMZ/MnO, and iRPPA@TMZ/MnO, determined by AAS. Data are expressed as mean ± SD (*n*= 3). **p< 0.01; ***p< 0.001.

The BBB is essential for regulating the homeostatic microenvironment of the CNS and is an inherent barrier that prevents most therapeutic agents to access the brain;^[^ ^13^, ^19^ ^]^ ≈95–98% of small molecular drugs and almost all macromolecular drugs cannot cross the BBB to enter the CNS.^[^ ^13^, ^19^ ^]^ Development of an effective strategy to overcome this challenge is of great significance for treating the CNS diseases such as gliomas.^[^ ^13b,c^ ^]^ Few recent studies have shown that the iRGD tumor‐targeting and penetrating cyclic peptide may help nanodrugs to cross the BBB and accumulate in gliomas by specifically binding to the *α*_v_ *β*_3_ integrin and NRP‐1 overexpressed in the endothelium of BBB and glioma cells.^[^ ^9^, ^14^ ^]^ We used an in vitro BBB model (**Figure** **3**a) based on the transwell system to investigate the targeting and BBB‐penetrating ability of iRGD. To visualize the nanodrug migration and cell uptake under the confocal laser scanning microscopy (CLSM), coumarin instead of TMZ was loaded into the nanodrug. As shown in Figure 3b, the C6 glioma cells cultured in the bottomed well showed markedly weak green fluorescence at 12 h after the addition of the non‐targeting nanodrug (PPA@coumarin/MnO) into the upper well, thereby indicating that it could not migrate much to the bottom well because of the BBB. However, after the addition of the targeting nanodrug (iRPPA@coumarin/MnO) into the upper well, the C6 glioma cells cultured in the bottom well exhibited markedly strong green fluorescence at 12 h, thereby indicating that the nanodrug could pass through the BBB‐mimicking endothelial monolayer and could be taken up by the glioma cells via endocytosis. Quantitative analysis using flow cytometry confirmed the result (Figure 3c). The integrity of BBB model after the studies was evaluated. The transendothelial electrical resistance (TEER) values before adding PPA@TMZ/MnO and iRPPA@TMZ/MnO were 248.2 ± 5.1 and 244.5 ± 7.4 Ω cm^2^, respectively. After removal of the nanodrug, the values of TEER were 235.7 ± 4.2 and 226.2 ± 9.5 Ω cm^2^, respectively, meaning a recovery to approach the normal value and indicated that the nanoparticle did not obviously destroy BBB. The results suggested that iRGD targeting could enable the nanodrug to cross the BBB and increase its internalization in C6 glioma cells.

A C6 glioma cell spheroid model was established further to evaluate the tumor‐penetrating effect of iRGD‐modified nanoparticles. C6 glioma cell spheroids with diameters of 250–400 µm were cultured with iRPPA@coumarin/MnO and PPA@coumarin/MnO for 12 h. As shown in Figure 3d, the cell spheroids cultured with iRPPA@coumarin/MnO showed a strong green fluorescence signal. In contrast, only weak green fluorescence signals were detected in the cell spheroid cultured with PPA@coumarin/MnO. TEM observations also revealed the difference in the levels of cell uptake between iRPPA@TMZ/MnO and PPA@TMZ/MnO. MnO encapsulation enabled easy identification of the intracellular nanodrug. As shown in Figure 3e, more nanoparticle clusters representing nanodrugs were found in the cytoplasm when C6 glioma cells were cultured with iRPPA@TMZ/MnO compared to PPA@TMZ/MnO. In vitro cellular MRI images showed an increased signal intensity of T1WI, while a decreased T1 value and T2 value on MRI after C6 glioma cells incubated with PPA@TMZ/MnO and iRPPA@TMZ/MnO (Figure S6, Supporting Information). The atomic absorption spectroscopy (AAS) analysis confirmed these results, i.e., the intracellular Mn content was significantly increased when C6 glioma cells were cultured with iRPPA@TMZ/MnO compared to PPA@TMZ/MnO (Figure 3f). We conclude that iRGD modification is likely to enhance tumor penetration and cell uptake of the nanodrugs. To further demonstrate the iRGD‐mediated targeting, the arginine‐glycine‐aspartic acid (RGD) competition study was performed by using quantitative flow cytometry analysis. As shown in Figure S7, Supporting Information, iRPPA@coumarin/MnO incubation resulted in obviously less coumarin fluorescence‐positive cells when the cells were pre‐treated with RGD. As RGD peptides specially bind to the *α*_v_ *β*_3_ integrin,^[^ ^20^ ^]^ the above result implied that iRGD introduced to the surface of nanodrug may mediate a special binding to *α*_v_ *β*_3_ integrin to enhance uptake by glioma cell.

**Figure 4:**
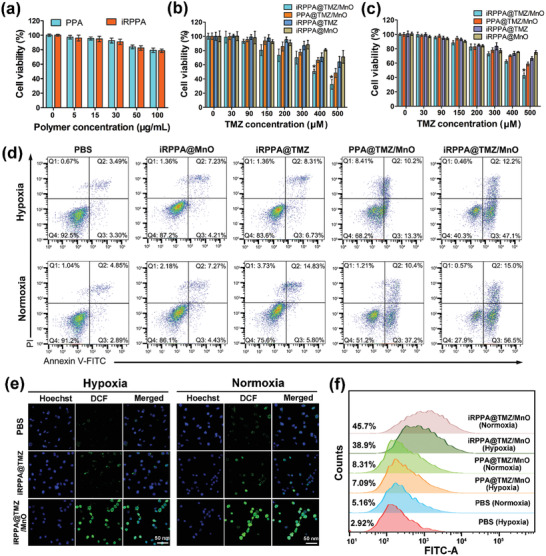
In vitro cytotoxicity and ROS generation. a) Cell viabilities of BCECs incubated with PPA and iRPPA at different concentrations (*n*= 6). Viabilities of C6 glioma cells after 24 h of incubation with different nanodrugs under the b) normoxic or c) hypoxic conditions. Data are expressed as mean ± SD (*n*= 6). *p< 0.05. d) Cell apoptosis detected by flow cytometry at 24 h after various cell incubations under the hypoxic or normoxic condition. e) In vitro cellular ROS generation of different nanodrugs under hypoxic or normoxic conditions. f) Quantitative flow cytometry analysis of the DCF fluorescence in C6 cells after different treatments under hypoxic or normoxic conditions.

We assessed the cytotoxicity of our nanodrugs in both normal and tumor cells in vitro using the cell counting kit‐8 (CCK8) analysis and luminescence‐based CellTiter‐Glo assay. As shown in **Figure** **4**a, brain capillary endothelial cells (BCECs) exhibited slightly decreased viabilities when incubated with drug/MnO‐free PPA and iRPPA even at concentrations up to 100 µg mL^–1^, thus demonstrating negligible cytotoxicity of both nanocarriers. The cytotoxicity of iRPPA@MnO, iRPPA@TMZ, PPA@TMZ/MnO, and iRPPA@TMZ/MnO was explored in C6 glioma cells by 24 h of incubation under both normoxic (Figure 4b; Figure S8, Supporting Information) and hypoxic conditions (Figure 4c; Figure S8, Supporting Information). Concentration‐dependent cytotoxic effects of iRPPA@MnO, iRPPA@TMZ, PPA@TMZ/MnO, and iRPPA@TMZ/MnO were detected in C6 glioma cells. Cells treated with TMZ‐loaded nanodrugs including iRPPA@TMZ, PPA@TMZ/MnO, and iRPPA@TMZ/MnO showed much higher viabilities under hypoxic conditions than under normoxic conditions, thereby indicating hypoxia‐induced resistance to TMZ treatment. iRPPA@TMZ/MnO incubation showed higher cytotoxicity to C6 glioma cells compared to that observed with iRPPA@TMZ incubation, suggesting a synergistic therapeutic effect of chemotherapy and chemodynamic therapy (CDT). Quantitative assessment of C6 glioma cell apoptosis using flow cytometry yielded consistent results. As shown in Figure 4d, the untreated cells exhibited an extremely low apoptosis rate under both hypoxic and normoxic conditions. In contrast, C6 glioma cells incubated with iRPPA@TMZ, PPA@TMZ/MnO, and iRPPA@TMZ/MnO showed an increased apoptosis rate, especially under normoxic conditions. The same trend was observed in the C6 glioma cell‐cycle arrest after various treatments under normoxic and hypoxic conditions (Figure S9, Supporting Information). C6 glioma cells treated with iRPPA@TMZ, PPA@TMZ/MnO, and iRPPA@TMZ/MnO induced increased cell cycle arrest at the G2/M phase both under normoxic and hypoxic conditions compared with those treated with PBS and iRPPA@MnO. This result implied that only the TMZ‐containing nanodrugs could generate an efficient G2/M phase cell cycle arrest.

CDT is an emerging therapeutic strategy for cancer treatment.^[^ ^12^, ^21^ ^]^ It depends on the in situ Fenton or Fenton‐like reaction to convert endogenous H_2_O_2_ into extremely toxic ·OH that can kill tumor cells.^[^ ^21a^ ^]^ Typically, ferrous ions (Fe^2+^) are the widely reported CDT agents that catalyze the conversion of the endogenous H_2_O_2_ into ·OH in tumors to trigger the ferroptosis of cancer cells. Recently, a self‐reinforcing CDT nanoagent based on MnO_2_ was developed.^[^ ^12^ ^]^ A Fenton‐like reaction was observed to deplete intracellular GSH and to generate ·OH radicals within the tumor effectively for inhibition of tumor growth.^[^ ^12^ ^]^ In this study, the ·OH‐generating ability of iRPPA@TMZ/MnO through a Fenton‐like reaction was investigated using methylene blue (MB), that is specifically degraded by ·OH (Figure S10, Supporting Information). At neutral pH, iRPPA@MnO did not affect the MB absorbance even in the presence of 100 × 10^−6^ m H_2_O_2_ but a significant decrease in MB absorbance was detected at pH 6.5 + 100 × 10^−6^ m H_2_O_2_. Additionally, iRPPA@MnO and MnCl_2_ showed similar effects on MB absorbance at pH 6.5 + 100 × 10^−6^ m H_2_O_2_. These results suggested that MnO embedded in the nanodrug underwent a Fenton‐like reaction in TME‐mimicking conditions to generate ·OH. ROS in C6 glioma cells were also detected using 2′,7′‐dichlorofluorescein diacetate (DCFH‐DA) ROS fluorescence probe under normoxic and hypoxic conditions (Figure 4e). Nonfluorescent DCFH‐DA can be oxidized into a fluorescent 2′,7′‐dichlorofluorescein (DCF) in the presence of ROS. CLSM images showed weak green fluorescence in the control cells and cells incubated with iRPPA@TMZ. In contrast, strong green fluorescence was observed in cells incubated with iRPPA@TMZ/MnO. C6 glioma cells under normoxic conditions exhibited slightly stronger green fluorescence than those under hypoxic conditions. As determined by quantitative analysis using flow cytometry, there were 38.5% of the ROS‐positive cells present under hypoxic conditions and 45.6% under normoxic conditions when C6 glioma cells were incubated with iRPPA@TMZ/MnO for 12 h, both levels being significantly higher than those in the control cells and cells receiving iRPPA@TMZ (Figure 4f). These data demonstrate that an efficient amount of ROS can be produced inside cells incubated with iRPPA@TMZ/MnO that is apparently due to the conversion of the endogenous H_2_O_2_ into ·OH via a Fenton‐like reaction.

**Figure 5:**
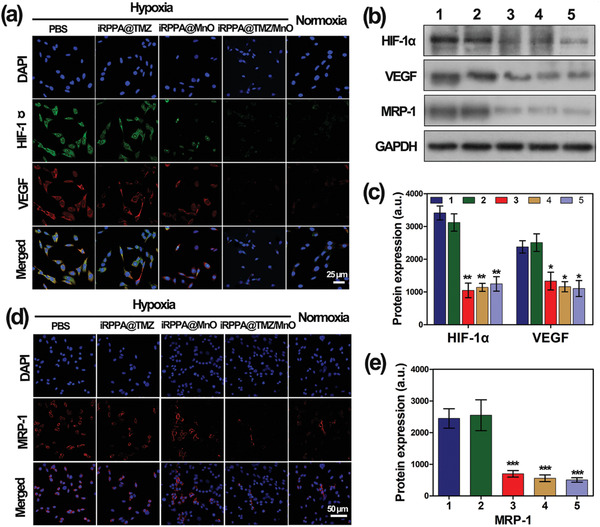
In vitro fluorescence expression of hypoxia‐related and drug resistance‐related biomarkers. a) Representative CLSM images showing HIF‐1*α*and VEGF expressions in C6 cells after different treatments under hypoxic or normoxic conditions. b) Western blot analysis showing protein expression of HIF‐1*α*, VEGF, and MRP‐1 in C6 cells after different treatments under hypoxic or normoxic conditions. c) Quantification of protein expression levels of HIF‐1*α*and VEGF using Western blot analysis. d) Representative CLSM images of MRP‐1 expression in C6 cells after different treatments under hypoxic and normoxic conditions. e) Quantification of protein expression levels of MRP‐1 using Western blot analysis. 1) PBS under hypoxic condition; 2) iRPPA@TMZ under hypoxic condition; 3) iRPPA@MnO under hypoxic condition; 4) PPA@TMZ/MnO under hypoxic condition; and 5) control cells under normoxic condition. Data are expressed as mean ± SD (*n*= 3). *p< 0.05; **p< 0.01; ***p< 0.001.

Hypoxia is a characteristic feature of the TME in various solid tumors that is caused mainly by an imbalance between oxygen supply and oxygen consumption.^[^ ^4^, ^22^ ^]^ HIF‐1*α* is a crucial transcription factor responding to hypoxia and can be highly expressed in tumor cells in the hypoxic microenvironment.^[^ ^22^, ^23^ ^]^ The VEGF is one of the HIF‐1*α*‐regulated proteins and is also commonly overexpressed in cancer cells to mediate hypoxia‐driven angiogenesis.^[^ ^23^ ^]^ Alleviation of tumor hypoxia could downregulate the expression of HIF‐1*α* and VEGF and inhibit tumor progression, invasion, metastasis, and angiogenesis.^[^ ^24^ ^]^ The expression levels of HIF‐1*α* and VEGF in C6 glioma cells treated with iRPPA@TMZ/MnO in vitro were assessed by CLSM observations and Western blot analysis. As shown in **Figure** **5**a, the expression levels of HIF‐1*α* and VEGF in tumor cells were markedly high under hypoxic conditions. However, the levels were significantly decreased in tumor cells incubated with iRPPA@MnO or iRPPA@TMZ/MnO under hypoxic conditions. Western blot analysis yielded similar results, thus supporting the conclusion that HIF‐1*α* and VEGF levels were significantly downregulated in C6 glioma cells treated with iRPPA@MnO or iRPPA@TMZ/MnO under hypoxic conditions (Figure 5b,c). These results lend further support to the notion that iRPPA@MnO and iRPPA@TMZ/MnO may effectively alleviate C6 cell hypoxia by releasing O_2_ under TME‐mimicking conditions.

Hypoxia contributes to tumor resistance against various treatments including chemotherapy, radiotherapy, phototherapy, immunotherapy, and CDT therapy.^[^ ^4^, ^25^ ^]^ It has been reported that hypoxia can induce the resistance of glioma to TMZ and that alleviation of tumor hypoxia can reverse glioma resistance to TMZ.^[^ ^7^, ^26^ ^]^ The multidrug resistance‐associated protein 1 (MRP‐1) is a transmembrane glycoprotein in the adenosine triphosphate (ATP)‐binding superfamily that has been found to associate with multidrug resistance via a mechanism of ʺpumpingʺ the cytotoxic drugs out of cells in an ATP‐dependent manner.^[^ ^27^ ^]^ It was also reported that hypoxia may aggravate chemoresistance in tumor cells by stimulating MRP‐1 expression.^[^ ^28^ ^]^ Alleviation of hypoxia could decrease the expression of MRP‐1 and result in enhanced sensitivity of glioma cells to chemotherapy. We determined the expression of MRP‐1 in C6 glioma cells by CLSM and Western blot analyses under hypoxic and normoxic conditions. As shown in Figure 5d, intensive red fluorescence (i.e., MRP‐1) was observed in C6 glioma cells under hypoxic conditions. However, red fluorescence was significantly reduced when the cells were incubated with iRPPA@MnO under hypoxic conditions, thereby approaching the level in cells treated with iRPPA@TMZ/MnO under normoxic conditions. Western blot analysis generated consistent and supportive results (Figure 5e). These results confirmed that iRPPA@MnO and iRPPA@TMZ/MnO could effectively reverse tumor hypoxia and decrease the expression level of MRP‐1 in glioma cells to enhance their chemotherapeutic sensitivity.

### 2.4 In Vivo Imaging of Nanodrug Distribution

**Figure 6:**
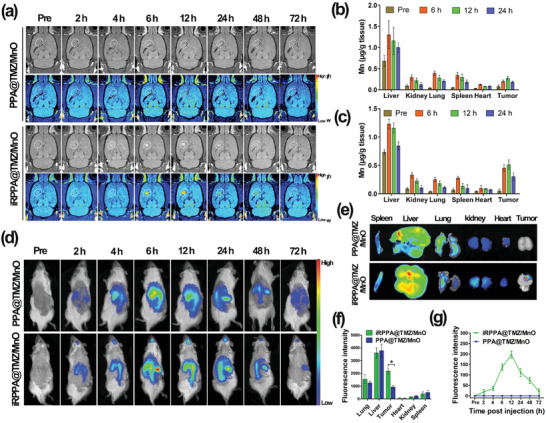
In vivo distribution of nanodrug. a) In vivo serial T1WI MRI images of the intracranial orthotopic glioma before and after intravenous injection of PPA@TMZ/MnO and iRPPA@TMZ/MnO (dose: 5 mg Mn kg^−1^). Mn accumulation in tumor and major organs before and after intravenous injection of b) iRPPA@TMZ/MnO and c) PPA@TMZ/MnO detected by ICP‐MS (*n*= 3). d) In vivo fluorescence imaging of C6 tumor‐bearing rats after intravenous injection of PPA@DiR/MnO and iRPPA@DiR/MnO at different time points. e) Ex vivo fluorescence imaging of the major organs and tumor at 72 h after nanodrug injection. f) Relative fluorescence intensities of the ex vivo major organs and tumor at 72 h after nanodrug injection. Data are expressed as mean ± SD (*n*= 3). *p< 0.05. g) Dynamic changes of fluorescence intensity in tumor against time after injection of PPA@DiR/MnO and iRPPA@DiR/MnO. Data are expressed as mean ± SD (*n*= 3).

MnO_2_/MnO‐based contrast agents have attracted much attention as an alternative to the widely used Gd‐based contrast agents that can induce serious nephrogenic systemic fibrosis (NSF) in patients with renal dysfunction.^[^ ^9^, ^17^ ^]^ MnO_2_/MnO‐based contrast agents can also react in the low‐pH TME enriched with H_2_O_2_ and GSH to release the MRI T1 contrast agent Mn^2+^ and to enable tumor‐specific detection with high imaging accuracy and sensitivity.^[^ ^9^, ^17^ ^]^ Unlike Gd‐based contrast agents that can induce gadolinium deposition in specific brain structures,^[^ ^29^ ^]^ the harmless water‐soluble Mn^2+^ can be rapidly cleared through the kidneys to avoid long‐term toxicity that makes it suitable for in vivo applications. To explore the potential of iRPPA@TMZ/MnO as a TME‐activatable MRI contrast agent, serial T1WI MRI images were acquired before and after the injection of iRPPA@TMZ/MnO in the tail vein (**Figure** **6**a; Figure S11, Supporting Information). The MRI signal intensity of the intracranial glioma was similar to that of the adjacent brain parenchyma on T1WI before nanodrug injection. It gradually increased to reach a peak at 12 h after injection and then slowly declined to the basal level at 72 h after injection. Compared with rats receiving PPA@TMZ/MnO, rats receiving iRPPA@TMZ/MnO showed more evident contrast enhancement for glioma, although both showed efficient contrast enhancement for glioma after intravenous injection at the same dose (5 mg Mn kg^−1^). These findings indicate that more MnO can cross the BBB to accumulate in the orthotopic glioma when assisted by the iRGD peptide. Although MnO is not a potent MRI contrast agent, MnO accumulated at the tumor site can be reduced to Mn^2+^ that may offer a significantly stronger MRI T1 contrast capacity. Indeed, the neurotoxicity of manganese is a concern in its clinical application.^[^ ^30^ ^]^ In comparison with the direct administration of free Mn^2+^, our iRGD‐targeted nanodrug mainly accumulated in the orthotopic glioma site, thereby decreasing the unwanted neurotoxicity to the CNS. This notion was supported by the fact that the Mn^2+^‐enhanced MRI signals were not detected in normal CNS tissue other than glioma.

We examined the in vivo distribution of Mn in tumors and major organs including the liver, kidneys, lungs, spleen, and heart at different post‐injection time points using inductively coupled plasma mass spectrometry (ICP‐MS). Before injection and at 6, 12, and 24 h after injection, the intratumoral Mn concentrations in PPA@TMZ/MnO (Figure 6b) and iRPPA@TMZ/MnO (Figure 6c) groups were 0.07, 0.19, 0.27, and 0.18 µg Mn g^−1^ tissue and 0.05, 0.45, 0.51, and 0.30 µg Mn g^−1^ tissue, respectively. iRPPA@TMZ/MnO exhibited a much better tumor accumulation than that exhibited by PPA@TMZ/MnO probably because iRGD modification could enhance the penetration of nanodrugs into orthotopic gliomas. iRPPA@TMZ/MnO and PPA@TMZ/MnO showed similar distribution patterns in major organs. In particular, a distinct accumulation of Mn in the liver was observed for both nanodrugs that was in line with other reports that stated nanoparticle systems tended to accumulate in the liver because of the uptake by Kupffer cells.^[^ ^31^ ^]^

We used fluorescence imaging to determine the nanodrugs in vivo distribution after injection in the tail vein. The near infrared (NIR) dye, 1,1′‐dioctadecyl‐3,3,3′,3′‐tetramethyl indotricarbocyanine iodide (DiR), was embedded in the nanocarrier together with MnO to make the nanoparticles visible for fluorescence imaging. After intravenous injection of iRPPA@DiR/MnO, the DiR fluorescence intensity in the tumor area increased gradually to reach the highest level at 12 h after injection and then slowly declined. Only a weak DiR fluorescence signal was detectable in the tumor area at 72 h after intravenous injection. No DiR fluorescence signal was detectable in the orthotopic tumor site up to 72 h after injection of PPA@DiR/MnO (Figure 6d). Obvious DiR fluorescence signals were detected in the liver after injection of iRPPA@DiR/MnO (68 ± 12 nm) or PPA@DiR/MnO (66 ± 11 nm). This phenomenon was in line with a great number of researches that nanoparticles with similar sizes tended to accumulate in the liver because of the uptake by Kupffer cells, the specialized macrophages in the liver.^[^ ^16^, ^31^, ^32^ ^]^ Ex vivo images of major organs and tumors were further analyzed 72 h after nanodrug injection and showed a preferential uptake by livers and tumors (Figure 6e,f). The dynamic changes of DiR fluorescence signals with post‐injection time were in line with the MRI analyses. This shows that the addition of the iRGD peptide effectively assisted nanodrugs to cross the BBB and penetrate orthotopic gliomas, resulting in enhanced intratumoral accumulation of nanodrugs.

### 2.5 In Vivo Therapeutic Effect and Biosafety of Nanodrugs in Orthotopic Glioma

**Figure 7:**
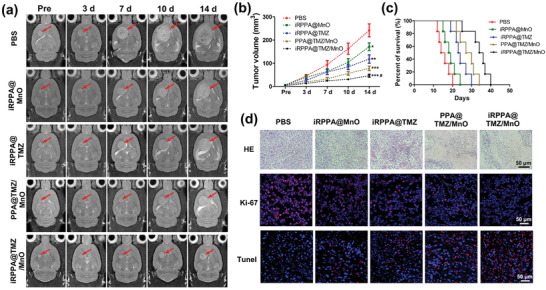
In vivo synergistic antitumor effects in orthotopic glioma‐bearing rats. a) Serial T2WI MRI images of orthotopic glioma (red arrows) within 14 days after treatment with PBS, iRPPA@MnO, iRPPA@TMZ, PPA@TMZ/MnO, and iRPPA@TMZ/MnO. Data are expressed as mean ± SD (*n*= 6), *p< 0.05; **p< 0.01; ***p< 0.001 versus PBS group,**^#^** *p*< 0.05 for iRPPA@TMZ/MnO group versus other nanodrug groups. b) Tumor volumes of orthotopic glioma for each group of tumor‐bearing rats. c) Survival rates of orthotopic glioma‐bearing rats (*n*= 6). d) In vivo HE, Ki‐67, and TUNEL analyses of orthotopic glioma at 14 days after different treatments for each group.

The in vivo antitumor efficacy of different nanodrugs was evaluated in a rat model of orthotopic glioma. The tumor volumes of orthotopic gliomas were dynamically monitored for 14 days using a clinical 3.0 T MRI scanner (**Figure** **7**a). As shown in Figure 7b, the tumor volume increased significantly over time in the PBS group, and iRPPA@MnO and iRPPA@TMZ showed moderate inhibitory effects on tumor growth. In contrast, PPA@MnO/TMZ and iRPPA@MnO/TMZ showed better inhibitory effect on tumor growth, thereby indicating that both chemotherapy and CDT were involved, with iRPPA@MnO/TMZ exhibiting the most effective tumor inhibition via the synergistic effect of the two therapies. The survival rates of rats receiving different treatments were in line with tumor growth (Figure 7c). The medium survival times of tumor‐bearing rats in the PBS, iRPPA@MnO, iRPPA@TMZ, PPA@TMZ/MnO, and iRPPA@TMZ/MnO groups were 15, 18.5, 23.5, 28.5, and 35 days, respectively. All rats died within 20 days of receiving PBS injections. The animals survived much longer when they received iRPPA@MnO, iRPPA@TMZ, PPA@TMZ/MnO, or iRPPA@TMZ/MnO, with iRPPA@TMZ/MnO treatment demonstrating the best effect in prolonging the survival time in rats.

Histological hematoxylin and eosin (H&E) staining assay showed a high density of cancer cells with small areas of necrosis tumor tissue sections of animals that received PBS injections. A decreased density of tumor cells and increased necrotic areas were observed in tumor tissue sections of animals that received nanodrug treatments. Treatment with iRPPA@TMZ/MnO showed the lowest density of tumor cells and the largest necrotic areas (Figure 7d). Immunofluorescence staining of Ki‐67 and terminal deoxynucleotidyl transferase biotin‐dUTP nick end labeling (TUNEL) assays were performed to assess the proliferation and apoptosis in tumor tissues of all animal groups. As shown in Figure 7d, abundant Ki‐67‐positive cells and few TUNEL‐positive cells were observed in the tumor tissue sections of rats receiving PBS injections, whereas the fewest Ki‐67‐positive cells and the most TUNEL‐positive cells were displayed in the tumor tissue sections of rats that received the iRPPA@TMZ/MnO treatment. These results demonstrated that TMZ and MnO incorporated into the nanodrug exhibited a strong synergistic anticancer effect in orthotopic glioma.

**Figure 8:**
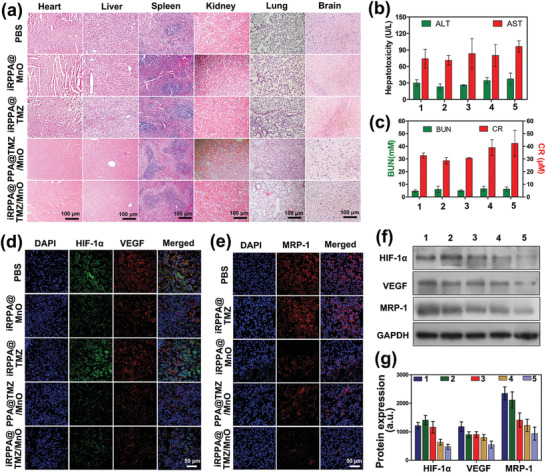
In vivo biosafety, alleviation of tumor hypoxia, and inhibition of MRP‐1 expression. a) Images of major organs stained by H&amp;E in each group receiving different treatments. b) ALT and AST blood biochemistry tests for liver function assessment in tumor‐bearing rats in each group (*n*= 3). c) CR and BUN blood biochemical analysis for the renal function assessment for each group of tumor‐bearing rats (*n*= 3). In vivo expressions of d) HIF‐1*α*and VEGF and e) MRP‐1 in tumor tissues of glioma‐bearing rats observed on CLSM. f) HIF‐1*α*, VEGF, and MRP‐1 protein expression in tumor tissues of glioma‐bearing rats analyzed by Western blot analysis. e) Quantification of protein expression levels of HIF‐1*α*, VEGF, and MRP‐1 based on Western blot analysis in tumor tissues. Data are expressed as mean ± SD (*n*= 3). 1) PBS; 2) iRPPA@TMZ; 3) iRPPA@MnO; 4) PPA@TMZ/MnO; and 5) iRPPA@TMZ/MnO.

The biosafety of drugs is the primary concern for their clinical applications. Long‐term systemic side effects of our nanodrugs were evaluated in vivo using histological and blood analysis. After 14 days of various treatments, H&E staining of the liver, heart, lung, kidney, and spleen tissues exhibited no pathological changes in any of the groups (**Figure** **8**a). Rats showed no evident weight loss in any group (Figure S12, Supporting Information). The serum biochemical markers of aspartate transaminase (AST), alanine aminotransferase (ALT), creatinine (CRE), and urea nitrogen (BUN) were within the normal range for all groups. No evident differences were detected between the different treatments (Figure 8b,c), thus indicating negligible hepatic and renal toxicities of the nanodrugs.

### 2.6 Alleviation of Tumor Hypoxia and Inhibition of MRP1 Expression In Vivo

Alleviation of tumor hypoxia in vivo and reversal of therapeutic resistance induced by tumor hypoxia were further evaluated by immunofluorescence staining and Western blot analyses for HIF‐1*α*, VEGF, and MRP‐1 expression. Immunofluorescence staining showed that PBS injections resulted in the highest expression levels of HIF‐1*α*, VEGF, and MRP‐1 in the orthotopic glioma tissue of rats, and the iRPPA@TMZ treatment showed similar results (Figure 8d,e). Much lower expression levels of HIF‐1*α*, VEGF, and MRP‐1 were detected in the tumor tissue of rats that received iRPPA@MnO, PPA@TMZ/MnO, and iRPPA@TMZ/MnO. Western blotting results were consistent with this finding (Figure 8f,g), thus showing the lowest expression levels of HIF‐1*α*, VEGF, and MRP‐1 in the iRPPA@TMZ/MnO‐treatment group. iRPPA@TMZ/MnO treatment may be effective in alleviating tumor hypoxia to overcome resistance to chemotherapy and CDT and further improve the synergistic anticancer effect.

## 3 Conclusion

The BBB may block the access of drugs and imaging agents to the CNS and present the greatest impediment for both the diagnosis and treatment of CNS‐associated diseases including brain glioma. In the present study, a TME‐responsive theranostic nanodrug iRPPA@TMZ/MnO capable of crossing the BBB and penetrating the orthotopic brain glioma was developed. Once accumulated in the orthotopic glioma, the nanodrug responded to the TME conditions simultaneously to release TMZ, Mn^2+^, and O_2_. The released TMZ and Mn^2+^ offered remarkable benefits for the growth inhibition of orthotopic glioma through a synergistic anticancer effect of chemotherapy and CDT. Moreover, the in situ‐produced O_2_ alleviated the tumor hypoxia and reduced the glioma resistance to anticancer therapy, thus further enhancing the therapeutic outcome. The released Mn^2+^ can also enable highly site‐specific MRI for tumor detection and noninvasive monitoring of the nanodrug‐treatment process. The study documented the potential of a novel theranostic nanodrug for MRI diagnosis and treatment of brain glioma.

## 4 Experimental Section

##### Materials

Manganese acetylacetonate (Mn(acac)3), dodecyl mercaptan, 18‐crown‐6‐ether, 2‐(diisopropylamino)ethyl methacrylate, 4‐dimethylaminopyridine, 1‐[3‐(dimethylamino)propyl]‐3‐ethylcarbodiimide hydrochloride, and TMZ were purchased from Merck Chemical Company (Germany). Functionalized PEG (MW = 2000) and iRGD peptide (sequence: c(CRGDKGPDC)) were purchased from 3A Chemicals (China). Oleic acid, oleic amine, benzyl ether, carbon disulfide, hydrochloric acid, azodiisobutyronitrile, chloroform, and phosphate buffer were purchased from J&K Scientific Company (China). Antibodies against HIF‐1*α*, VEGF, MRP‐1, TUNEL, and Ki‐67 for immunofluorescence staining and Western blot analysis were obtained from Abcam (Shanghai, China).

##### Synthesis of Polymers and Preparation of Nanodrugs

Oleic acid‐coated manganese monoxide nanoparticles were prepared via a solvothermal reaction. Briefly, Mn(acac)_3_ (1.06 g, 3.0 mmol), oleic acid (3.3 mL, 9.0 mmol), oleic amine (3 mL, 9.0 mmol), and 1,2‐hexadecanediol (2.8 g, 10 mmol) were dissolved in 30 mL of benzyl ether and heated at 110 °C under vacuum for 30 min to remove the water. The solution was heated at 200 °C for 3 h and then rapidly heated to 300 °C for 1 h. The obtained nanocrystals were refluxed under a flow of argon, cooled to room temperature, precipitated with ethanol, and centrifuged at 6000 rpm to generate the oleic MnO nanoparticles.

PDPA, a pH‐sensitive building block, was synthesized according to the method described in a previous study.^[^ ^16^ ^]^ To prepare PEG‐PDPA, dried methoxy polyethylene glycol (mPEG) (0.94 g, 0.47 mmol), PDPA (1.3 g, 0.47 mmol), 1‐ethyl‐3‐(3‐dimethylaminopropyl)carbodiimide hydrochloride (EDC HCl) (0.13 g, 0.7 mmol), and 4‐dimethylaminopyridine (DMAP) (0.011 g, 0.094 mmol) were dissolved in 20 mL dichloromethane and stirred for 24 h at room temperature. The solution was then precipitated in ethanol. To prepare iRGD‐PEG‐PDPA, iRGD‐*N*‐hydroxysuccinimide (iRGD‐NHS) (0.5 mmol) was first mixed with HOOC‐PEG‐NH_2_ (0.35 mmol) for 3 days to obtain iRGD‐PEG‐NH_2_. PDPA (1.3 g, 0.47 mmol) was mixed with EDC HCl (0.13 g, 0.7 mmol) and NHS (0.12, 0.1 mmol) in chloroform for 1.5 h to obtain PDPA‐NHS; iRGD‐PEG‐NH_2_ (0.95 g, 0.50 mmol) was then added into the solution and stirred for 3 days. The solution was precipitated in ethyl ether. The precipitate was dissolved in a NaCO_3_ solution (0.5 m) and stirred for 2 h for acetyl‐group deprotection. The solution was dialyzed to remove NaAc and then freeze‐dried to obtain iRGD‐PEG‐PDPA. Finally, iRGD‐PEG‐PDPA or PEG‐PDPA was dissolved in chloroform and mixed with oleic acid‐coated MnO/TMZ in dimethylsulfoxide (DMSO) and the mixture was sonicated using an ultrasonic oscillator. Chloroform was removed by evaporation and DMSO by dialysis. To visualize the nanodrugs for CLSM and in vivo fluorescence imaging, coumarin or DiR was loaded into the nanodrugs to replace TMZ. Briefly, coumarin or DiR was dissolved in chloroform/DMSO and mixed with the iRPPA solution. Then, the solution was sonicated, and the organic solvent was removed by dialysis to allow the formation of micelle.

##### Characterization of Polymer and Nanodrugs

For TEM, a JEM‐1400 Plus (JEOL, Japan) at a voltage of 120 kV was used. SAED images of MnO nanoparticles were obtained using TEM with the probe aligned at certain selected areas of the sample. Magnetic properties of the oleic MnO nanoparticles and iRPPA@MnO were assessed using a magnetic hysteresis loop using PPMS‐9T magnetic property measurement at 10 and 300 K. The ^1^H NMR spectra of iRGD‐PEG‐PDPA and mPEG‐PDPA were measured using an Ascend 400 (Bruker). XRD spectra of MnO nanoparticles were recorded on the Rigaku D/MAX 2200 vpc X‐ray diffractometer (CuK*α* radiation *λ* = 1.54056) at 40 kV and 40 mA. The hydrodynamic diameter and zeta potential of iRPPA, PPA, iRPPA@MnO, iRPPA@TMZ, and iRPPA@TMZ/MnO were measured using DLS on the Nano‐ZS90 Zetasizer (Malvern).

The responsiveness of nanodrugs to the TME was examined by determining morphological changes; the release of O_2_, Mn^2+^, and TMZ; and *r*_1_ magnetic relaxation properties of nanodrugs under different stimulations. The morphological changes of iRPPA@MnO and iRPPA@TMZ/MnO under pH 6.5 + 100 × 10^−6^ m H_2_O_2_ were analyzed using TEM. The O_2_ concentrations were measured with a portable dissolved oxygen meter (Taizhou Leici Instrument Equipment Co. Ltd., Taizhou, China). Different concentrations of iRPPA@TMZ/MnO were added to H_2_O_2_ solutions at pH 6.5 + 0 – 100 × 10^−6^ m, and the O_2_ concentrations were recorded at different time intervals. The produced Mn^2+^ was measured using inductively coupled plasma optical emission spectrometry (ICP‐OES, Varian 710‐ES, USA). Briefly, a dialysis bag (MWCO:14 kDa) containing 1.5 mL of a solution of PPA@TMZ/MnO or iRPPA@TMZ/MnO (5 mg mL^–1^) was placed in Na_2_HPO_4_/NaH_2_PO_4_ buffer under pH 7.4, or pH 6.5+H_2_O_2_. At predesigned time points, 2 mL of buffer was replaced with the fresh buffer to measure Mn^2+^. The TMZ release profile of PPA@TMZ/MnO and iRPPA@TMZ/MnO under different conditions was measured by HPLC (Shimadzu Nexara XR) at time intervals using the same method as that for Mn^2+^ measurement. The *r*_1_ and *r*_2_ values of iRPPA@TMZ/MnO and PPA@TMZ/MnO under different stimulations were measured using a clinical 3.0 T MRI scanner (Philips Achieva, Philips Medical Systems, The Netherlands) with an eight‐channel sense knee coil.^[^ ^9c^ ^]^

##### In Vitro BBB‐Crossing Efficiency, Tumor Penetration, and Cell Uptake

BCECs and C6 glioma cells were purchased from Cyagen Bioscience Technology Co. (Guangzhou, China). The cells were cultured in the Dulbecco's modified Eagle's medium (DMEM, Gibco, NY, USA) supplemented with 10% FBS (Gibco, USA) and 1% streptomycin–penicillin (Gibco, USA) at 37 °C in a humidified atmosphere of 5% CO_2_ and 21% O_2_. For cellular hypoxia experiments, C6 glioma cells were cultured under hypoxic conditions (2% O_2_) for 48 h.

An in vitro two‐compartment BBB model was employed to detect the ability of iRGD‐modified nanoparticles to cross the BBB. Briefly, 5 × 10^4^ cells BCECs were seeded into the transwell upper chamber with a polycarbonate filter (1.0 mm pore size) and cultured for 7 days to reach a TEER above 200 Ω cm^2^. Then, iRPPA@coumarin/MnO and PPA@coumarin/MnO were added to the upper well and incubated for 12 h. The C6 cells in the bottom well were stained with lysotracker (Thermo Fisher Scientific, Waltham, MA, USA) for 1 h and then with 4′,6‐diamidino‐2‐phenylindole (DAPI) (1 mg mL^–1^) for 1 min. The cellular uptake of coumarin‐labeled nanodrugs by C6 cells in the bottom well was observed using CLSM (LSM710; Carl Zeiss, Jena, Germany) and quantified by flow cytometry (FACSCalibur; BD Biosciences, Franklin Lakes, NJ, USA). To further demonstrate the *α*_v_ *β*_3_ integrin‐mediated cell uptake of the iRGD‐modified nanodrug, the RGD competition study was performed by using the quantitative flow cytometry analysis. Briefly, the C6 cells were pre‐treated with RGD for 6 h, then iRPPA@coumarin/MnO or PPA@coumarin/MnO was added to incubate the cells for 12 h. The cell uptake of coumarin‐labeled nanodrugs by C6 cells was quantified by flow cytometry (FACSCalibur).

To evaluate the tumor‐penetrating ability of iRGD‐modified nanodrugs, a C6 glioma cell spheroid was established. Briefly, a round‐bottom ultralow attachment 96‐well spheroid microplate (Corning, 4520, USA) was first coated with 15% matrigel (Corning). Then, a single cell suspension of C6 cells with a density of 1 × 10^4^ cells per well was added to each well. After centrifugation at 1000 rpm for 10 min, the cells on the microplate were incubated at 37 °C in a 90% humidified incubator with 5% CO_2_ for 3–5 days to form the C6 cell spheroids of 250–400 µm in diameter. The spheroids were then incubated with iRPPA@coumarin/MnO or PPA@coumarin/MnO for 12 h. After washing twice with PBS, the spheroids were stained with DAPI and observed on CLSM.

To further detect the Mn accumulation in C6 cells, in vitro cellular TEM, MRI, and AAS were performed. For cellular TEM, after incubation with iRPPA@TMZ/MnO or PPA@TMZ/MnO for 12 h, the C6 cells were fixed in 2.5% glutaraldehyde cacodylate buffer at 4 °C overnight and then treated with 1% OsO_4_ for 1 h. The C6 cells were dehydrated, embedded, and cut into ultrathin sections (60 nm). The sections of C6 cells were observed using a TEM (Hitachi H‐7500, Tokyo, Japan). For in vitro MRI, C6 glioma cells were incubated with iRPPA@TMZ/MnO or PPA@TMZ/MnO for 12 h, harvested, and embedded in 200 µL of 2% agarose gel (Invitrogen, Merelbeke, Belgium) in a 96‐well plate, and scanned on a clinical 3.0 T MRI with the same sequences and parameters as described above. For AAS, after incubation with iRPPA@TMZ/MnO or PPA@TMZ/MnO for 12 h, the C6 cells were added to the 1 m HCl solution. After Mn was thoroughly released from the cells and dissolved, it was quantified by AAS.

##### In Vitro Cell Toxicity

In vitro cell viability was determined using the CCK‐8 assay and the luminescence‐based CellTiter‐Glo assay. Briefly, cells were seeded in a 96‐well plate at a density of 5 × 10^3^ cells per well. After treatment with different nanodrugs at different concentrations for 24 h, the cells were incubated with 10 µL of CCK‐8 solution (Kumamoto, Japan) in each well at 37 °C for 2 h or 100 µL of CellTiter‐Glo reagent in each well at 37 °C for 10 min. The CCK‐8 assay and CellTiter‐Glo assay were then performed according to the manufacturer's instructions. The in vitro cell apoptosis rate and cycle distribution were detected by flow cytometry analysis. After incubation with iRPPA@MnO, iRPPA@TMZ, PPA@TMZ/MnO, iRPPA@TMZ/MnO, or PBS for 24 h under hypoxic or normoxic conditions, cells were double‐stained with annexin V‐FITC/propidium iodide (PI) for cell apoptosis detection. After treatment with different nanodrugs, C6 glioma cells were fixed in chilled 70% ethanol for 24 h, resuspended in PBS containing RNase A for 30 min at 37 °C, and incubated with PI for 30 min at 4 °C for cell cycle analysis. All experiments were performed in triplicate.

##### In Vitro Detection of ·OH

The in vitro ·OH‐generating ability of iRPPA@TMZ/MnO was evaluated using a bleaching experiment with MB. Briefly, 10 µg mL^−1^ MB was dispersed in different buffers (pH 6.5, pH 7.4+H_2_O_2_, and pH 6.5+H_2_O_2_), and then iRPPA@TMZ/MnO was added. The absorption intensity after MB degradation was measured at 655 nm on an LS 55 fluorescence spectrometer (PerkinElmer) using a 1 cm path‐length cuvette. MnCl_2_ solution supplemented with pH 6.5+H_2_O_2_ served as a positive control. After determining the generation of ·OH in aqueous solution, the production of intracellular ·OH in C6 cells was further analyzed using DCFH‐DA fluorescence staining. After treatment with iRPPA@TMZ or iRPPA@TMZ/MnO for 12 h under hypoxic (2% O_2_) or normoxic (21% O_2_) conditions, C6 cells were stained with DCFH‐DA (10 × 10^−6^ m) for 20 min at 37 °C. The green fluorescence of DCF within C6 cells was immediately observed on CLSM and quantitatively analyzed using flow cytometry as described above.

##### In Vitro Immunofluorescence Assay and Western Blot Analysis

The in vitro expression levels of HIF‐1*α*, VEGF, and MRP‐1 in C6 glioma cells after incubation with different formulations were assessed using immunofluorescence assay and Western blot analysis. C6 glioma cells were incubated with PBS, iRPPA@TMZ, iRPPA@MnO, or iRPPA@TMZ/MnO under hypoxic conditions for 24 h, and C6 glioma cells under normoxia were used as the positive control. For the immunofluorescence assay, C6 glioma cells were incubated in sequence with primary antibodies against HIF‐1*α*, VEGF, and MRP‐1 at 37 °C for 2 h and a secondary antibody at 37 °C for 1 h, and then stained with DAPI for 1 min. Fluorescence intensity was observed under CLSM. For Western blot analysis, the treated C6 glioma cells were added to a modified RIPA lysis buffer (Thermo Fisher Scientific, Rockford, IL) containing a protease inhibitor cocktail (Thermo Fisher Scientific). The protein samples (20 µg) were separated by 12% sodium dodecyl sulphate–polyacrylamide gel electrophoresis (SDS–PAGE) and then transferred onto polyvinylidene difluoride (PVDF) membranes. Protein blots were probed with primary antibodies against HIF‐1*α*, VEGF, and MRP‐1 overnight at 4 °C, followed by incubation with horseradish peroxidase‐conjugated secondary antibodies for 1 h at 37 °C. Glyceraldehyde‐3‐phosphate dehydrogenase (GADPH; 1:10 000; KangChen, Shanghai, China) served as a protein loading control.

##### Animal Model

All animal experimental procedures were performed in accordance with the Guidelines for Care and Use of Laboratory Animals of Sun Yat‐sen University (SYSU) and approved by Animal Care and Use Committee of SYSU (no. SYSU‐IACUC‐2019‐B868). All experiments involving animals strictly followed the Institutional Animal Care and Use Committee Guidebook, 2002 (https://olaw.nih.gov/guidance/ARENA-OLAW-IACUC-guidebook.htm). Adult male Wistar rats (weighing 220–250 g) were obtained from the Animal Experiment Centre of Sun Yat‐sen University and maintained in a standard animal facility with standard food and ad libitum water. The rat orthotopic glioma model was established according to the methods described in a previous study.^[^ ^29^ ^]^ Briefly, 5 × 10^5^ C6 glioma cells were stereotaxically injected into the left striatum. When the intracranial tumors grew to about 2–3 mm in maximal diameter confirmed by MRI, rats were used for further in vivo experiments.

##### In Vivo Biodistribution and MRI

The intracranial tumor‐bearing rats were injected with 1 mL of PPA@DiR/MnO or iRPPA@DiR/MnO via the tail vein at a dose of 1 mg DiR kg^−1^ body weight. The fluorescent images were obtained using an in vivo imaging system (Carestream IS 4000, USA) before injection and at 2, 4, 6, 12, 24, 48, and 72 h after injection. At 72 h post‐injection, rats were sacrificed to obtain the major organs (heart, lungs, liver, spleen, and kidneys) and tumors for ex vivo imaging. To detect the dynamic changes in MRI signal intensity, an in vivo MRI study was performed on a clinical 3.0 T MR system (Achieva; Philips Medical Systems). After injection of PPA@TMZ/MnO or iRPPA@TMZ/MnO via the tail vein at a dose of 2 mg Mn kg^−1^ body weight, the tumor‐bearing rats were imaged at various time intervals by using the MRI unit with the same parameters as used in a previous study.^[^ ^33^ ^]^ The distribution and accumulation of Mn deposits in major organs and tumors were further detected by ICP‐MS. Briefly, before and 6, 12, and 24 h after injection of PPA@TMZ/MnO or iRPPA@TMZ/MnO, the major organs (heart, lungs, liver, spleen, and kidneys) and tumors were harvested, dissolved in a mixture of 30% hydrogen peroxide solution and nitric acid, and digested using a microwave system. The Mn content was then measured by ICP‐MS (iCAP RQ, Thermo Fisher Scientific, USA).

##### In Vivo Synergistic Anticancer Efficiency

For therapeutic assays, the intracranial tumor‐bearing rats were randomly divided into five groups (*n* = 12 in each group) to receive intravenous injection of PBS, iRPPA@MnO, iRPPA@TMZ, PPA@TMZ/MnO, or iRPPA@TMZ/MnO (4.5 mg TMZ kg^–1^ body weight or 9 mg Mn kg^–1^ body weight) every 2 days for a total of 10 injections. Before injection and at 1, 3, 7, 10, and 14 days after injection, MRI was performed on a clinical 3.0 T unit to measure the tumor volumes of the brain orthotopic glioma. The acquisition sequences included axial and coronal fast spin‐echo T2‐weighted imaging (T2WI) with the following acquisition parameters: repetition time (TR) = 800 ms, echo time (TE) = 60 ms, field of view (FOV) = 60 mm, matrix = 256 × 256, and section thickness/gap = 1.0/0.0 mm. The tumor volumes were calculated by manually drawing the region of interest (ROI) of tumor areas on each slice using the ImageJ software (NIH, Bethesda, MD, USA). All tumor area slices were then summed and multiplied by slice thickness to generate the tumor volumes.^[^ ^33^ ^]^ Additionally, the body weights and survival time of tumor‐bearing rats were recorded, and the survival curve was generated using the Kaplan–Meier method. The animals were euthanized when one of the following symptoms occurred: circling, blindness, dementia, and convulsion.

##### Histology, Western Blot Analysis, and Biochemical Analysis

At 14 days after injection of different formulations, 15 tumor‐bearing rats were euthanized and the major organs (heart, liver, spleen, lungs, and kidneys) and tumors were quickly harvested. For histological analysis, the major organs and tumors were fixed in 4% buffered paraformaldehyde, embedded in optimum cutting‐temperature compound (OCT), and cut into 8 µm‐thick slices. The sections were then stained with H&E according to the standard protocols and observed under an optical microscope. For immunofluorescence, the tumor tissue sections were incubated with rabbit polyclonal Ki‐67 antibody, rabbit polyclonal TUNEL antibody, rabbit polyclonal HIF‐1*α*, rabbit polyclonal VEGF, or rabbit polyclonal MRP‐1 for 2 h at 37 °C, and then stained with a corresponding secondary antibody (Invitrogen, Carlsbad, CA, USA) and DAPI. Immunofluorescence images were observed using CLSM. For Western blot analysis, the tumors were collected and homogenized. Protein concentrations in the tissue homogenates were determined using BCA kits. The protein samples were then subjected to SDS–PAGE and transferred onto PVDF membranes for Western blot analysis as described above. To determine the toxic effects of nanodrugs, blood biochemical analysis (including ALT and AST for liver function assessment; CR and BUN for renal function assessment) of serum samples in tumor‐bearing rats was performed using the shared medical facilities and platforms at the Sun Yat‐sen Memorial Hospital.

##### Statistical Analysis

The data are presented as mean ± SD. Statistical significance was analyzed using the Student's *t*‐test or one‐way ANOVA (SPSS software, version 15.0, SPSS, Inc.), followed by the Bonferroni post hoc test for multiple pairwise comparisons. Survival curves were plotted by using the log‐rank test based on the Kaplan–Meier method. All statistical tests were two‐sided, and *p* < 0.05 was considered statistically significant.

## Conflict of Interest

The authors declare no conflict of interest.

## Supporting information

Supporting Information
