# Supplementary material for: Theranostic Nanomedicine for Synergistic Chemodynamic Therapy and Chemotherapy of Orthotopic Glioma
Source: Adv Sci (Weinh). 2020 Nov 13;7(24):2003036. doi: 10.1002/advs.202003036 (PMC7740078; doi:10.1002/advs.202003036)
Supplement: Supplementary file 1 — Supporting Information [file ADVS-7-2003036-s001.pdf]

© 2020 Wiley-VCH GmbH

ADVANCED SCIENCE
Open Access

Supporting Information

for *Adv. Sci.*, DOI: 10.1002/advs.202003036

Theranostic Nanomedicine for Synergistic Chemodynamic Therapy and Chemotherapy of Orthotopic Glioma

Junyi Tan, Xiaohui Duan, Fang Zhang, Xiaohua Ban, Jiaji Mao, Minghui Cao, Shisong Han, Xintao Shuai, Jun Shen**

Supporting Information

Theranostic Nanomedicine for Synergistic Chemodynamic Therapy and Chemotherapy of Orthotopic Glioma

Junyi Tan, Xiaohui Duan, Fang Zhang, Xiaohua Ban, Jiaji Mao, Minghui Cao, Shisong Han, Xintao Shuai, Jun Shen**

Dr. J. Tan, Dr. X. Duan, Dr. F. Zhang, Dr. J. Mao, Dr. M. Cao, Prof. X. Shuai, Prof. J. Shen

Department of Radiology, Sun Yat-Sen Memorial Hospital, Sun Yat-sen University, Guangzhou 510120, China.

E-mail: shuaixt@mail.sysu.edu.cn; shenjun@mail.sysu.edu.cn.

Dr. J. Tan, Dr. S. Han, Prof. X. Shuai

PCFM Lab of Ministry of Education, School of Material Science and Engineering

Sun Yat-Sen University

Guangzhou 510275, China

Dr. X. Duan, Dr. F. Zhang, Dr. J. Mao, Dr. M. Cao, Prof. J. Shen

Guangdong Provincial Key Laboratory of Malignant Tumour Epigenetics and Gene Regulation

Sun Yat-Sen Memorial Hospital, Sun Yat-sen University, Guangzhou 510120, China.

X. Ban

Department of Radiology, Sun Yat-sen University Cancer Centre, Sun Yat-sen University, Guangzhou 510060, China

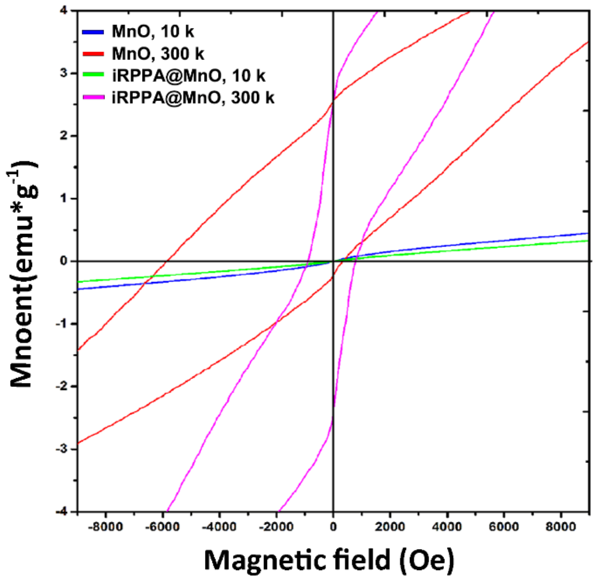

Figure S1. Hysteresis curves of MnO and iRPPA@MnO at 10 and 300 K.

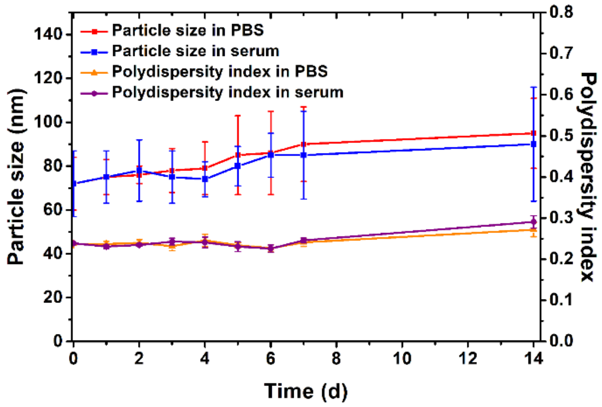

Figure S2. The stability of iRPPA@TMZ/MnO after diluted into PBS (pH=7.4), and 10% fetal bovine serum (FBS)-containing PBS. Data are expressed as mean ± SD (n=3).

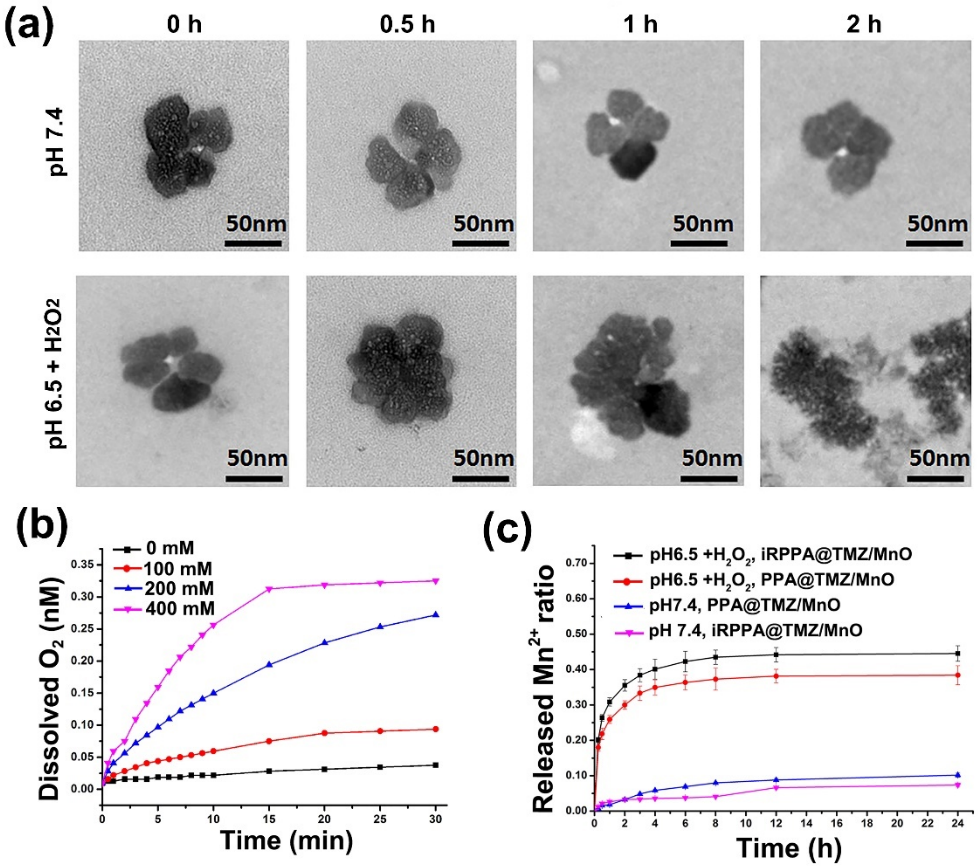

Figure S3. *In vitro* tumor microenvironment (TME) responsive properties of nanodrugs. (a) Serial TEM images showed the decomposition process of iRPPA@MnO at pH 7.4 or pH 6.5 + 100×10^{-6} H_2O_2. (b) Oxygen generation of iRPPA@TMZ/MnO at various concentrations (0~400 nM) at pH 6.5 + 100×10^{-6} M H_2O_2. (c) Mn^{2+} produced from PPA@TMZ/MnO and iRPPA@TMZ/MnO at pH 7.4 and pH 6.5 + 100×10^{-6} M H_2O_2. Data are expressed as mean ± SD (n=3).

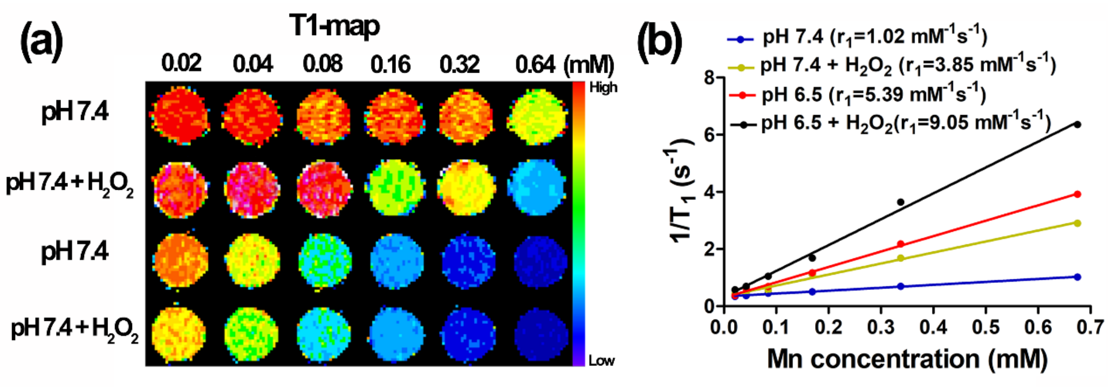

Figure S4. *In vitro* MRI T1-map (a) and r_1 relaxivities (b) of PPA@TMZ/MnO at different stimulations.

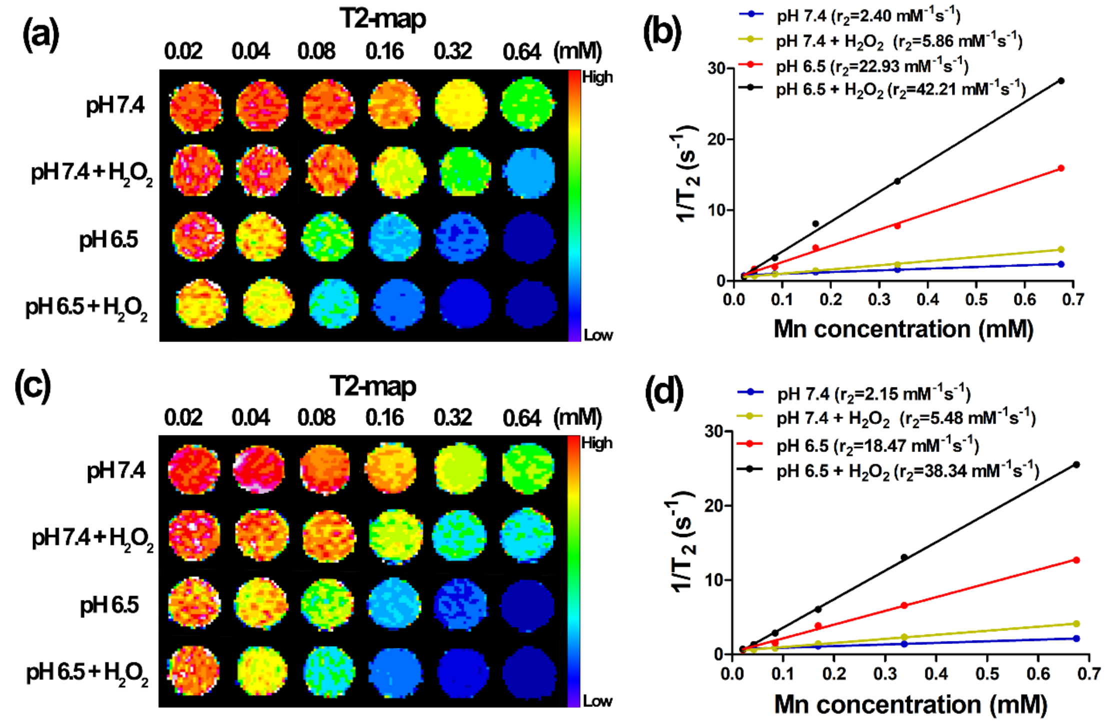

Figure S5. *In vitro* MRI T2-map (a) and r_2 relaxivities (b) of iRPPA@TMZ/MnO at different stimulations. *In vitro* MRI T2-map (c) and r_2 relaxivities (d) of PPA@TMZ/MnO at different stimulations.

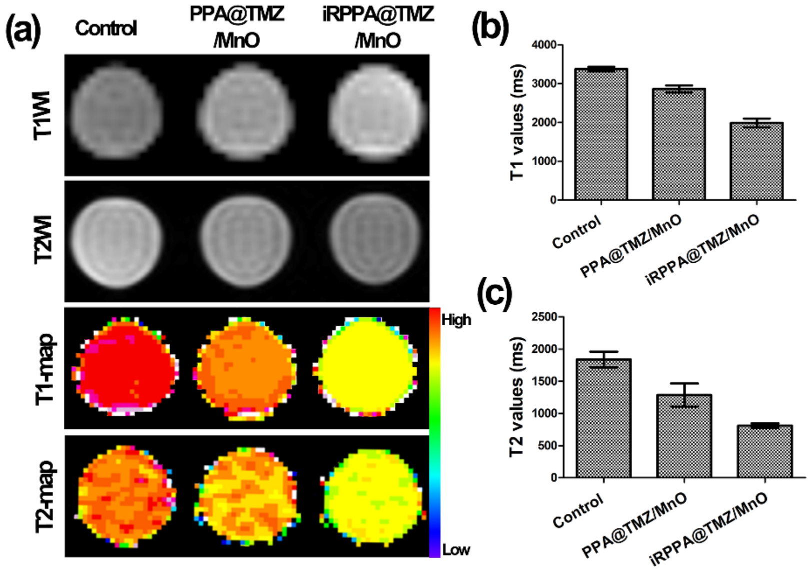

Figure S6. *In vitro* MR images (a), T1 value (b) and T2 value (c) of C6 glioma cells after treatment with PPA@TMZ/MnO and iRPPA@TMZ/MnO. Data are expressed as mean ± SD (n=6), * $P < 0.05$

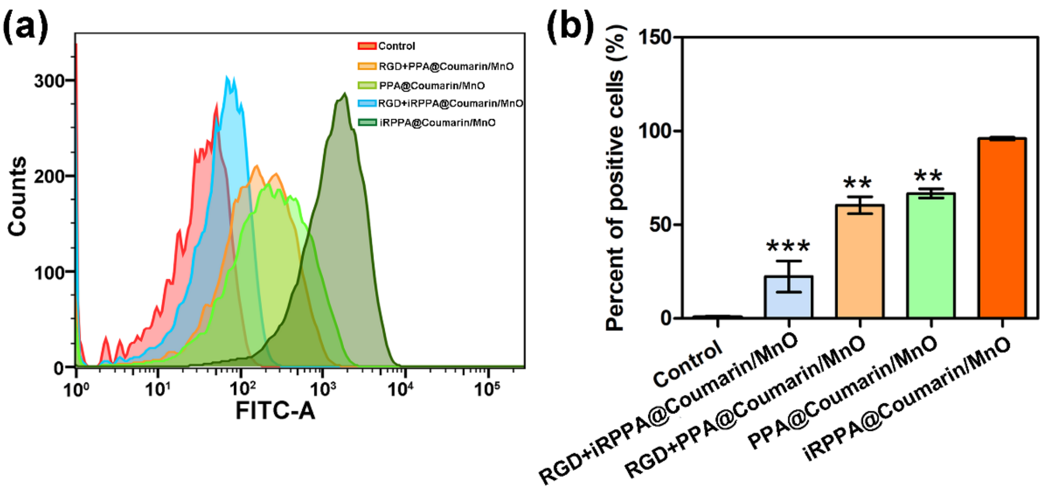

Figure S7. (a) Flow cytometric analysis shows the uptake of nanodrugs by C6 glioma cells after incubation with RGD + PPA@Coumarin/MnO, PPA@Coumarin/MnO, RGD + iRPPA@Coumarin/MnO or iRPPA@Coumarin/MnO. (b) Quantification of the

percentages of coumarin-positive C6 cells after different treatments. Data are expressed as mean ± SD (n=6), ** $P < 0.01$, *** $P < 0.001$ *vs* iRPPA@Coumarin/MnO.

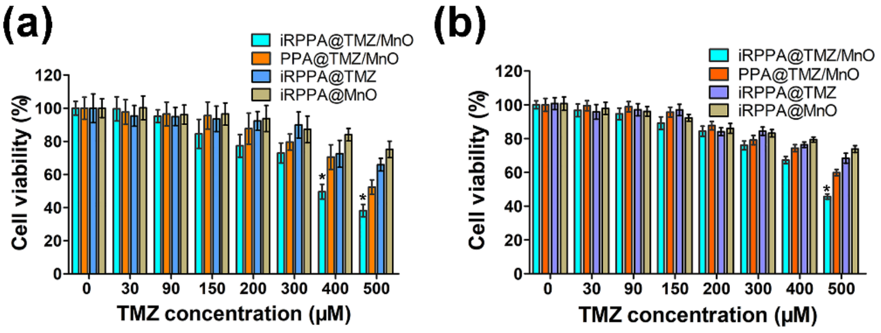

Figure S8. *In vitro* cytotoxicities detected by using the CellTiter-Glo assay. Cell viabilities of C6 glioma cells after 24 h incubation with different nanodrugs under the normoxic (a) or hypoxic conditions (b). Data are expressed as mean ± SD (n = 6). *$P < 0.05$.

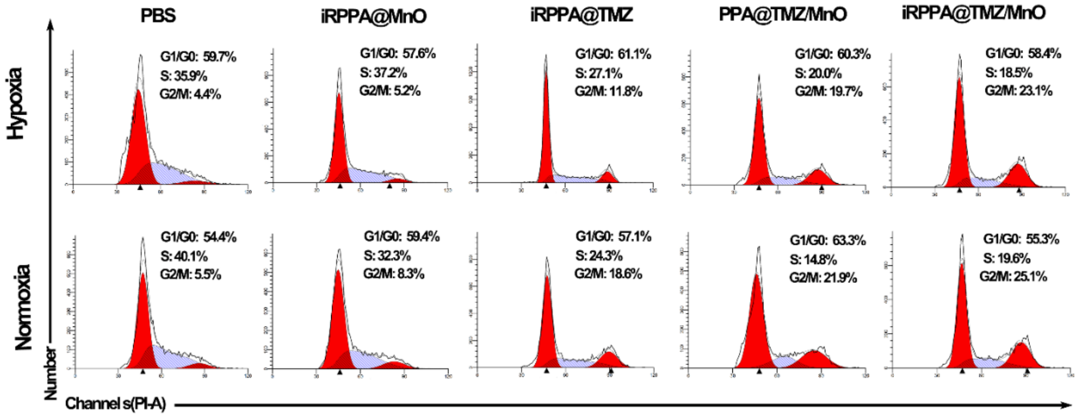

Figure S9. Cell cycle distribution of C6 glioma cells after 24 h incubation with different nanodrugs under the normoxic or hypoxic condition.

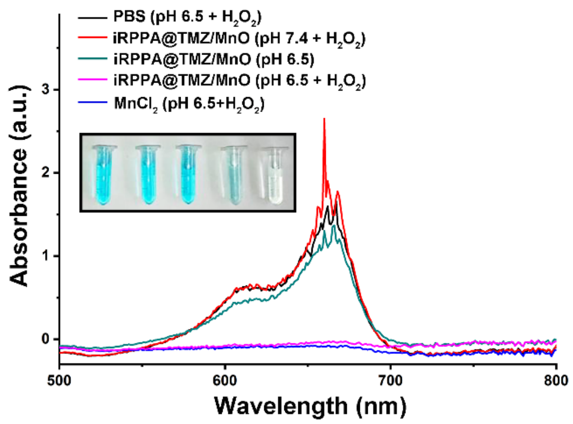

Figure S10. MB absorption spectra and photo (inset) after degradation by Mn^{2+}-mediated Fenton-like reaction in different solutions.

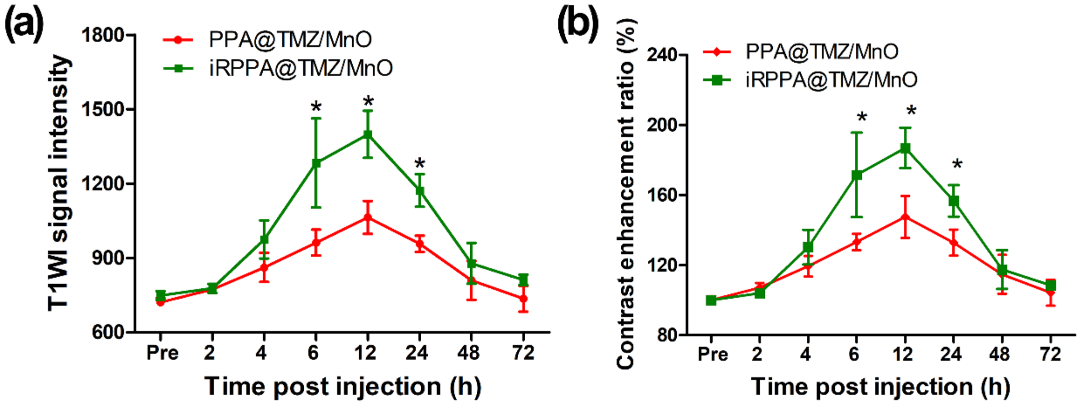

Figure S11. Dynamic changes of T1WI signal intensity and contrast enhancement ratio in orthotopic glioma against time after injection of PPA@DiR/MnO and iRPPA@DiR/MnO. Data are expressed as mean ± SD (n=3), * P<0.05.

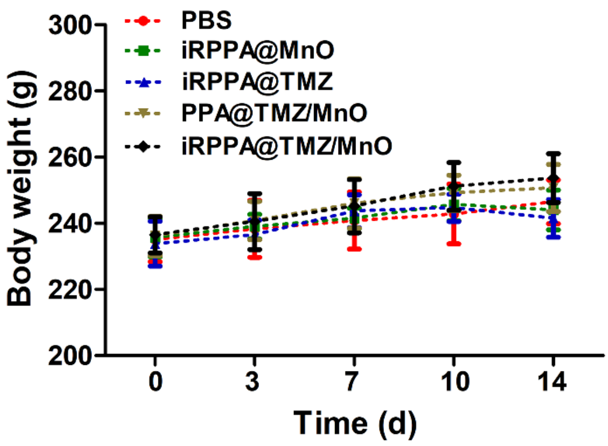

Figure S12. Dynamic changes of body weights of tumor-bearing rats receiving various treatments. Data are expressed as mean ± SD (n = 6).
